# Developmental Transcriptome Profiling of the Tibial Reveals the Underlying Molecular Basis for Why Newly Hatched Quails Can Walk While Newly Hatched Pigeons Cannot

**DOI:** 10.3389/fcell.2022.745129

**Published:** 2022-02-07

**Authors:** Qifan Wu, Hehe Liu, Qinglan Yang, Bin Wei, Luyao Wang, Qian Tang, Jianmei Wang, Yang Xi, Chunchun Han, Jiwen Wang, Liang Li

**Affiliations:** Farm Animal Genetic Resources Exploration and Innovation Key Laboratory of Sichuan Province, College of Animal Science and Technology, Sichuan Agricultural University, Chengdu, China

**Keywords:** precocial, altricial, embryonic development, locomotion, ossification

## Abstract

Birds can be classified into altricial and precocial species. The hatchlings of altricial birds cannot stand, whereas precocial birds can walk and run soon after hatching. It might be owing to the development of the hindlimb bones in the embryo stage, but the molecular regulatory basis underlying the divergence is unclear. To address this issue, we chose the altricial pigeon and the precocial Japanese quail as model animals. The data of tibia weight rate, embryonic skeletal staining, and tibia tissues paraffin section during the embryonic stage showed that the Japanese quail and pigeon have similar skeletal development patterns, but the former had a faster calcification rate. We utilized the comparative transcriptome approach to screen the genes and pathways related to this heterochronism. We separately analyzed the gene expression of tibia tissues of quail and pigeon at two consecutive time points from an inability to stand to be able to stand. There were 2910 differentially expressed genes (DEGs) of quail, and 1635 DEGs of pigeon, respectively. A total of 409 DEGs in common in the quail and pigeon. On the other hand, we compared the gene expression profiles of pigeons and quails at four time points, and screened out eight pairs of expression profiles with similar expression trends but delayed expression in pigeons. By screening the common genes in each pair of expression profiles, we obtained a gene set consisting of 152 genes. A total of 79 genes were shared by the 409 DEGs and the 152 genes. Gene Ontology analysis of these common genes showed that 21 genes including the *COL* gene family (*COL11A1, COL9A3, COL9A1*)*, IHH, MSX2, SFRP1, ATP6V1B1, SRGN, CTHRC1, NOG,* and *GDF5* involved in the process of endochondral ossification. These genes were the candidate genes for the difference of tibial development between pigeon and quail. This is the first known study on the embryo skeletal staining in pigeon. It provides some new insights for studying skeletal development mechanisms and locomotor ability of altricial and precocial bird species.

## Introduction

Locomotion is one of the most important ecological functions in animals. How can some mammals and birds locomote spontaneously soon after birth or hatching while other animals require weeks to months to do so? Early developmental heterochronies could, in some cases, be linked to dramatic differences in life histories, for example, when comparing species exhibiting an altricial condition at birth to precocial species. Such differences in developmental strategy are owing to the animals’ environmental suitability and evolutionary diversity during long-term evolution (Keyte and Smith, 2010; Augustine et al., 2019).

The early ontogeny of locomotion is a primary diagnostic feature in defining the altricial-precocial spectrum (Chen et al., 2019). It can be addressed on various levels, such as the developing states of the appendicular skeleton, muscles, and motor nerves in embryos (Kernell, 1998; Dial and Carrier, 2012). Experimental evidence suggests that postural constraints play an important role in preventing the early expression of locomotor behavior in altricial species (Gillian, 2000; Speake and Wood, 2005; Augustine et al., 2019). Compared with altricial species, the differentiation of motor neurons and muscle cells occurred earlier in precocial species (Charvet and Striedter, 2011). The coordinated movement patterns are not entirely innate, but that rapid neuromotor maturation, potentially also the result of the rearrangement or recombination of existing motor modules, takes place in these precocial animals (Vanden Hole et al., 2017).

Skeletons are the primary system that carries the body, and the degree of calcification of the hindlimbs in the embryonic stage plays a vital role in standing and locomoting (Pourlis et al., 1998). Species-specific developmental timing of skeletogenic events has gained attention in comparative developmental biology. There are tradeoffs in apportioning bone mass to the hindlimb and forelimb that could account for these patterns in locomotor behavior within the mallard (*Anas platyrhynchos*). At 30 days post-hatching, when body mass is 50% of adult values, bone growth (length and width) in the hindlimbs and forelimbs averages 90% and 60% of adult values, respectively (Dial and Carrier, 2012). Marsupials and placental animals have developmental metachronism related to ossification sequences. Specifically, relatively late ossification of hindlimb bones and early ossification of the anterior axial skeleton has been found in marsupials (Weisbecker et al., 2008). Avian species with higher growth rates tend to have a higher proportion of cartilage in their skeletons (Starck, 1996; Blom and Lilja, 2010).

Comparative embryological studies covering different taxa and different character complexes have gathered increasing evidence for intraspecific and interspecific variability in the timing of early embryogenic events (Sheil and Greenbaum, 2005; Wilson et al., 2010). Mitgutsch describes and analyzes the temporal order of ossification of skeletal elements in the one altricial bird (Zebra finch), and in the two precocial birds (Japanese quail and White Pekin duck), the proximal long bones of the limbs are among the first elements to ossify (Mitgutsch et al., 2011). The more distal elements of the limbs ossify comparatively late. The results show a general pattern largely concordant with those reported for other birds (Maxwell, 2008, 2009). Although domestic turkey, domestic chicken, and Japanese quail differ in size and incubation period, the three Galliformes examined here demonstrated remarkable consistency in their degree of ossification at hatching, as well as the identity of the ossified elements (Maxwell, 2008).

Domestic pigeons (*Columba livia* domesticus) and Japanese quail (*Coturnix japonica*) are typical altricial and precocial birds, respectively. Domestic pigeons belong to Columbiformes, and all species in this order are altricial birds. Domestic pigeon hatchlings can only perform alternating stepping movements. It usually takes half a month for a white king pigeon to stand and walk normally post-hatching. However, Japanese quail belongs to Galliformes, and all species in this order are precocial birds. Japanese quails can walk and run well within 24 h post-hatching (Augustine et al., 2019). The two species have similar incubation periods (16–18 days for quail and 18–20 days for pigeons) and egg sizes, making them ideal for studying altricial-precocial spectrum skeletal development.

This study characterized the differences in hindlimb skeletal development before and after hatching of pigeon and Japanese quail for the first time. And then, we analyzed the gene expression of the tibia using RNA-seq and explored the molecular mechanism regulating skeletal development in precocial and altricial birds.

## Materials and Methods

### Experimental Design and Sampling

Fertilized eggs of white king pigeon (*Columba*) and Japanese quail (*Coturnix japonica*) were collected within 6 h after laying and stored at 15°C. The eggs were provided by Yimeng Pigeon Company (Linyi, Shandong) and Youjia Fresh Food Company (Lianyungang, Jiangsu), respectively. Eggs were put into an incubator (BLF-440C, Chengdu, China) within 1 week of laying. The temperature and relative humidity of the incubator were adjusted to 38.0 ± 0.2°C and 60%, respectively. Through preliminary experiments, it was found that the incubation period of white king pigeon was 20 days and that of Japanese quail was 18 days. For 14 days post-hatching, pigeons could stand and walk normally.

**FIGURE 1:**
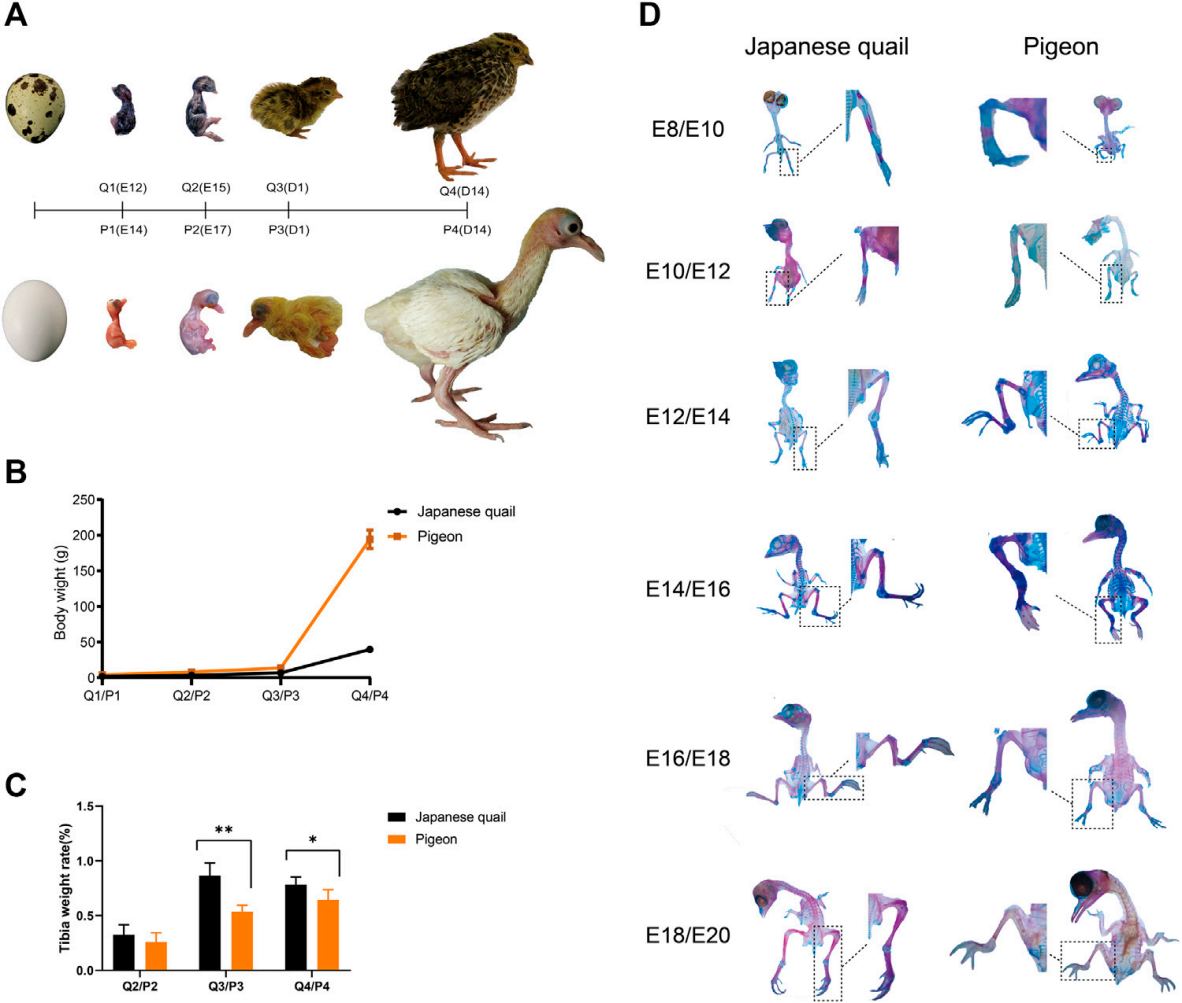
The development of pigeons and quails in the embryonic period and the early phase post-hatching. **(A)** The ontogeny of quail and pigeon before and after hatching. **(B)** The change of bodyweight of quail and pigeon in the four growth stages. The error bars on the graphs represent std. dev. **(C)** The change of tibia weight rate of quail and pigeon in the four growth stages. “∗∗” means the difference is significant at the 0.01 level, “∗” means the difference is significant at the 0.05 level. The error bars on the graphs represent std. dev. **(D)** Comparison of quail and pigeon skeletal staining at all six time points during the embryonic period. Q: quail, P: pigeon.

Therefore, quail embryos were used for skeletal staining at 8 (E8), 10 (E10), 12 (E12), 14 (E14), 16 (E16), and 18 (E18) days of incubation. Pigeon embryos were used for skeletal staining at 10 (E10), 12 (E12), 14 (E14), 16 (E16), 18 (E18), and 20 (E20) days of incubation. Three quails and pigeons were used at each time point, respectively. The bodyweight and tibia weight of each individual was recorded at four phases, which were 6 days before hatching (E12 of quail and E14 of pigeon, represented by Q1 and P1, respectively), 3 days before hatching (E15 of quail and E17 of pigeon, represented by Q2 and P2, respectively), the day of hatching (D1, quail and pigeon are represented by Q3 and P3, respectively) and 14 days after hatching (D14, quail and pigeon are represented by Q4 and P4, respectively) (Figure 1A). Six quails and pigeons were used at each time point, respectively. In addition, at each of the four phases, 3 quails (pigeons) of similar weight were selected, collecting both tibias, the left tibia was used for hematoxylin-eosin staining, and the right tibia was used for transcriptome analysis.

### Embryo Skeletal Staining

Skeletons of the embryos were stained with Alcian blue 8 GX and alizarin red S (Beijing Solarbio Science & Technology Co. Ltd., Beijing, China) for cartilage and ossified bones, respectively. The staining steps, which were modified from the methods of Dingerkus and Uhler (1977), Yoshifumi and Masaoki (1999) and Young et al. (2000) and listed in Supplementary Table S1. Observation of skeletons was performed under a dissecting microscope paying attention to the timing of chondrification and calcification.

### Paraffin Section

Tibia tissues were fixed in 4% paraformaldehyde for more than 24 h, then decalcified in 10% EDTA at room temperature for 9 weeks and embedded into paraffin. Serial sections (3 μm) were cut for the subsequent treatment. Hematoxylin-eosin staining was performed to visualize the changes in the tissues, the nucleus was stained in blue, and the cytoplasm was in red (Rigueur and Lyons, 2014). The sections took panoramic photos with 20× magnification using Digital Pathology Total Section Scanner (VS120-S6-W, Olympus, Japan).

### RNA Extraction and RNA-Seq

The tibia tissues were used as the material to extract total RNA. According to the manufacturer’s instructions, total RNA was isolated using Trizol reagent (Invitrogen, United States). Poly (A) + mRNA was purified with mRNA capture beads, and then the mRNA was randomly segmented into small fragments by divalent cations in a fragmentation buffer.

These short fragments were used as templates to synthesize the first-strand cDNA using random hexamer primers. Second-strand cDNA was synthesized using rnaseh and DNA polymerase I. Short cDNA fragments were purified with VAHTSTM DNA Clean Beads. The cNDA fragments were then connected with sequencing adapters according to an Illumina protocol. After agarose gel electrophoresis, the target fragments of 300–500 bp were selected for PCR amplification. The cDNA libraries were prepared using the VAHTS mRNA-seq V3 Library Prep Kit for Illumina® according to the product instructions (Jingjing et al., 2014; Jingjing et al., 2015). First, the quality and size of the cDNA libraries for sequencing were checked using the Agilent 2200 tapestation system (Agilent). Then, cDNA libraries were sequenced on the Illumina sequencing platform (Novaseq 6000 Illumina). Fastqc analyzed raw reads for quality, and high-quality reads with Q > 20 were obtained using ngstoolkits (version: 2.3.3) (Fumagalli et al., 2014). Finally, functions of the unigenes were annotated based on sequence similarities to sequences in the public UniProt database (Grabherr et al., 2011).

### Raw Data Processing

Low-quality reads were filtered using stringent criteria by FASTX (v 0.0.13): (1) reads with more than 50% of bases with quality <20; (2) the base quality is <10 at the 3’ ends of the reads; (3) reads with overrepresented adaptors; (4) reads having an “N” base; (5) reads shorter than 20 bp (Shengnan et al., 2019). Hisat2 (v2.1.0) was used to align the clean data to reference the genome of *Columba livia* (Refseq: GCF_000337935.1) and *Coturnix japonica* (Refseq: GCF_001577835.2) (Shapiro et al., 2013; Otake and Park, 2016). The mapped data was collated and formatted by Samtools. Then the output GTF files were merged into a single unified transcript using stringTie merge function (Meng et al., 2018). The merged transcripts were compared to the reference annotation using gffcompare program (v0.10.1, https://ccb.jhu.edu/software/stringtie/gffcompare.shtml) (Feibiao et al., 2019). The quantification of gene expression levels was performed as follows. The gene expression levels were estimated according to fragments per kilobase of transcript per million fragments mapped (FPKM). The reads count value was used for DEGs identification and the FPKM value was used for all other analyses. DEGs between the experimental groups were identified using the deseq2 R package. *p*-value <0.05 and |log2fc| > 1 were set as the screening criteria for significantly differential expression. SIMCA-P software (version 14.1, Umetrics, Umea, Sweden) was used for Principal Component Analysis (PCA) of transcriptome data (A et al., 2015).

### Gene Expression Pattern Analysis

The gene expression pattern was analyzed by Short Time-series Expression Miner (v1.3.13) software (Ernst and Bar-Joseph, 2006). The “Log normalize data” method was adopted in the strategy of expression quantity transformation. Other options were set to default since they have given optimal results with both biological and simulated data. The expression level used in this study was the previously calculated FPKM value. The p-value of the clustered profile was less than 0.05, which was considered significant (Zhongxian et al., 2018).

### GO and KEGG Pathway Enrichment Analysis

Functional enrichment analysis of differentially expressed genes was achieved using a GO analysis via DAVID v6.8 (https://david.ncifcrf.gov/home.jsp) (Huang et al., 2009). Pathway enrichment of candidate genes was achieved using KEGG enrichment via KOBAS 3.0.11 (http://kobas.cbi.pku.edu.cn/) (Xie et al., 2011). Chicken was used as a reference species. Q-value < 0.05 was considered significant. R package and related packages are used to realize data visualization.

### Phylogenetic Analysis

Phylogenetic tree was constructed using protein sequences, which were downloaded from NCBI. The numbers of the protein sequence were listed in supplemental file (Supplementary Table S2). The sequences of different species were aligned by ClustalW of MEGA7 (Sudhir et al., 2016). The tree was constructed by IQ-TREE (http://iqtree.cibiv.univie.ac.at/) using the Maximum Likelihood method (Trifinopoulos et al., 2016). The best-fit model JTTDCMut + I + G4 was chosen according to Bayesian Information Criterion using the ModelFinder method (Kalyaanamoorthy et al., 2017). The bootstrap consensus tree inferred from 1000 replicates was taken to represent the evolutionary history of the taxa analyzed (Felsenstein and Joseph, 1985). Initial tree for the heuristic search were obtained automatically by selecting the topology with superior log likelihood value. Using iTOL v6 (https://itol.embl.de/) to display and annotate the tree.

## Results

### Skeletal Development and Staining Analysis of Quail and Pigeon

We compared the body weight and tibia development of pigeon and quail during embryonic and early posthatching. In the early phase of embryonic development, the embryo weight of quail is heavier than that of pigeon. But from E12, pigeon embryos are heavier than quail embryos. Pigeons grow faster than quails after hatching (Figures 1A–C), indicating differences in embryonic development strategies between the two species. The tibia weight ratio of quails was higher than that of pigeons at the same stage (Figure 1C). At the time of hatching (Q3/P3), the difference of tibia weight rate between pigeon and quail was significant (*p* < 0.01), and at the 14th day of age (Q4/P4), the difference was significant (*p* < 0.05). The tibia weight rate of pigeons showed positive allometric growth from the late embryonic period to the 14th day after hatching (Figure 1C). Conversely, the Japanese quail has the highest tibia weight rate at hatching, exhibiting negative allometric growth after hatching (Figure 1C).

The staining results of embryonic skeleton of quail and pigeon are shown in Figure 1D. Chondrification was confirmed by blue color stained with Alcian blue 8GX and calcification by red color with alizarin red S. In terms of the overall skeleton of the embryo, quails have a higher percentage of alizarin red staining area than pigeons at the same embryonic stage (Figure 1D), indicating that the embryonic calcification degree of quail were higher than pigeon. Then, we focused on the calcification of the hindlimb bones. Only the midpoints of the tibia and femur of the pigeon were stained with alizarin red in E10, indicating that the primary ossification center had just occurred. However, more area was stained with alizarin red in the femur and tibia of the quail in E8, and the tarsometatarsus appeared red. The digits of the E10 quail appear in area stained with alizarin red, indicating the beginning of calcification, while the digit of the E12 pigeon was still blue and in a cartilage state. In the E12-16 (E14-18) of the quail (pigeon), most of the femur and tibia of quail and pigeon has beenstained with alizarin red, and only the joint was still in a cartilage state. In E18/E20, The femur and tibia have been completely calcified, and the area of pigeon digits stained with Alcian blue was still more than that of quail. In general, the quail has a higher calcification rate of hindlimb bones than pigeons in the embryonic stage.

### Tibia and Femur Comparison of Quail and Pigeon

**FIGURE 2:**
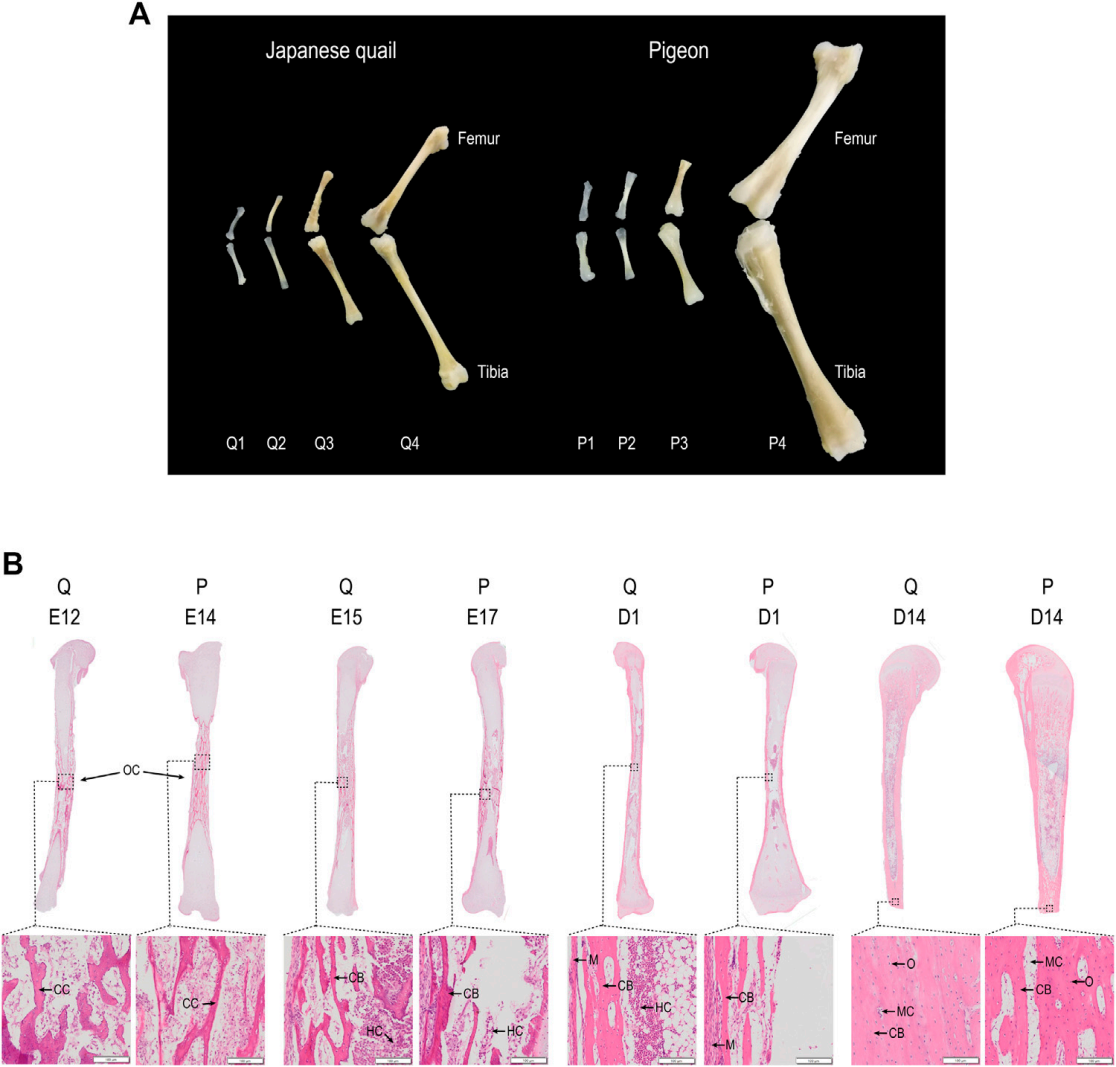
Femur and tibia development of quail and pigeon before and post-hatching. **(A)** Comparison of femur and tibia morphology in four growth stages of quail and pigeon. **(B)** Comparison of tibia panoramic photos in four growth stages of quail and pigeon. OC: ossification center; CC: calcified cartilage; HC: hematopoietic cells; CB: cortical bone; MC: marrow cavity; O: osteocyte; M: myocyte; Q: quail; P: pigeon.

We describe the four histological development stages of the tibia and femur from quail and pigeon (Figure 2A). The phenotypic data indicated that the developmental patterns of quail and pigeon tibia were similar. The primary ossification center appears in the middle of the tibia first, cartilage was absorbed and formed marrow cavity, and finally, the peripheral calcification was observed (Figure 2B). The calcification progresses from the middle of the tibia to both ends. Comparing the tibia tissues of quail and pigeon at the same time point, it was found that the percentage of calcified bone matrix area (CBM.Ar) of quail was higher than that of pigeon (Table 1). Conversely, the percentage of cartilage matrix area (CM.Ar) of quail was lower than that of pigeon (Table 1). It indicated that the development of quail tibia was more mature before and after hatching. The difference in tibia development between quail and pigeon was caused by heterochronism.

**TABLE 1:** Comparison of tibial morphological parameters between quail and pigeon.

	CM.Ar,%	CBM.Ar,%
Q1	26.44 ± 2.78^∗∗^	11.28 ± 2.54
P1	49.86 ± 5.54^∗∗^	6.64 ± 1.90
p*-value*	0.003	0.065
Q2	26.07 ± 5.60	8.17 ± 2.38
P2	35.75 ± 3.69	6.30 ± 0.79
p*-value*	0.067	0.267
Q3	16.28 ± 1.40^∗∗^	11.30 ± 1.68
P3	31.15 ± 2.14^∗∗^	7.64 ± 2.57
p*-value*	0.001	0.107
Q4	2.31 ± 0.60^∗∗^	33.60 ± 2.3^∗∗^
P4	7.83 ± 1.00^∗∗^	20.14 ± 2.07^∗∗^
p*-value*	0.001	0.002

CM.Ar: percent cartilage matrix area; CBM.Ar: percent calcified bone matrix area; “^∗∗^” means the difference is significant at the 0.01 level. Data are represented as Mean ± SD (n = 3).

### Overview of Transcriptome Dataset

A total of 24 cDNA libraries were synthesized, and transcriptome RNA-sequencing (RNA-seq) data was generated. The RNA-seq yielded a total of 537.95 M clean reads, and 160.24 Gb clean data, which were more than 93.29% of bases scoring Q30 (Supplementary Table S3). In addition, 86.83% and 73.78% of the clean reads of quail and pigeon were mapped in proper pairs with the reference genome *Columba livia* and *Coturnix japonica*, respectively (Supplementary Table S4). PCA showed that PC1 of the quail and pigeon transcripts accounted for 34.3% and 27.3% of the variation, respectively (Supplementary Figures S1A,B). The samples were clustered well within the group, which indicated that the repeatability is well and the quality of the sample is reliable.

### Differential Expressed Gene Analysis

**FIGURE 3:**
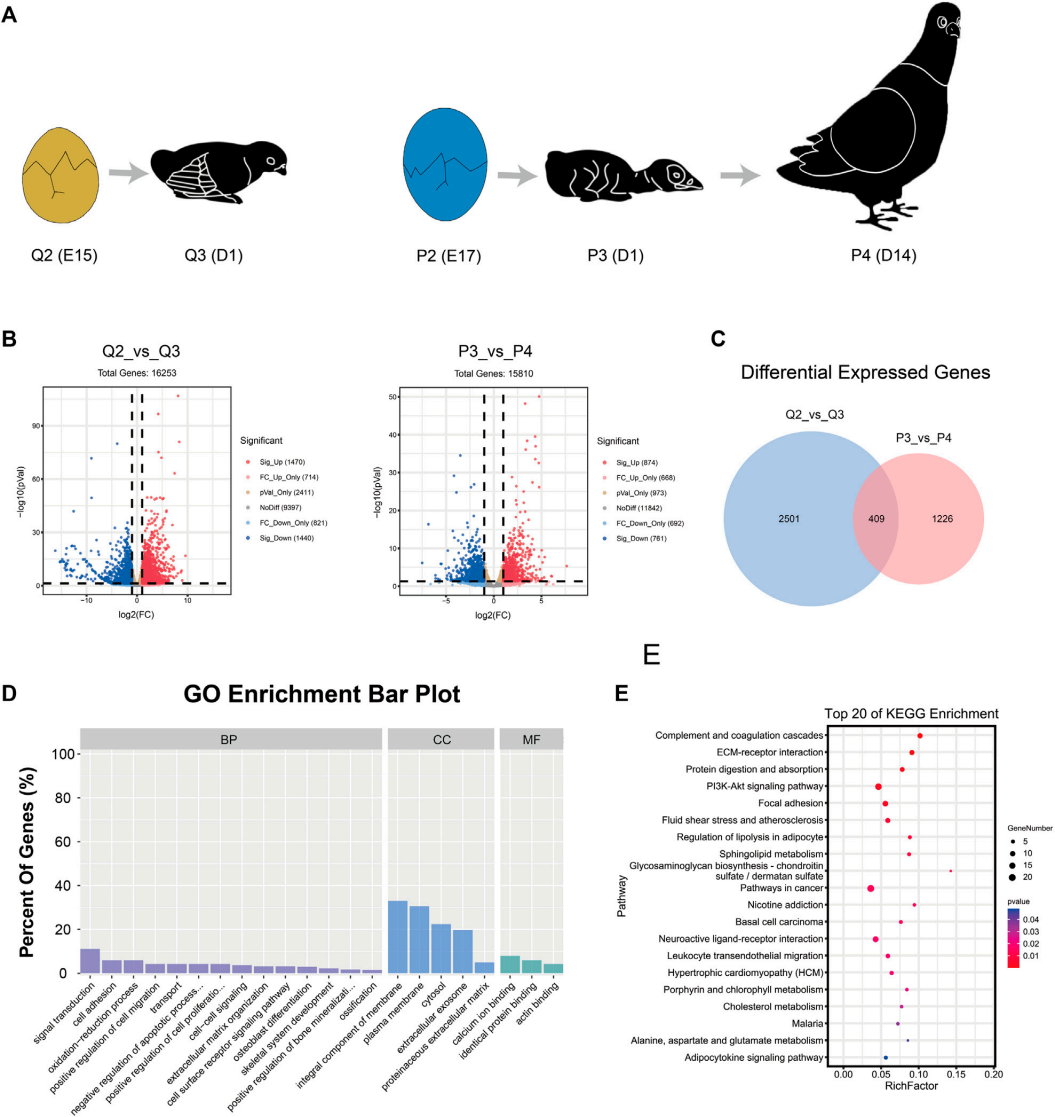
Analysis of differential expression genes in quail and pigeon. **(A)** The time for quail and pigeon from being unable to stand to be able to stand. **(B)** Volcano plots of genes in group Q2 vs. Q3 and P3 vs. P4. **(C)** The common DEGs between Q2 vs. Q3 and P3 vs. P4. **(D)** GO enrichment analysis of the shared DEGs. **(E)** KEGG pathway enrichment analysis of the shared DEGs. BP: biological process, CC: cellular component, MF: Molecular Function.

We compared gene expression in pigeons and quails before and after they were able to walk (Figure 3A). Q2 vs. Q3 has 2910 DEGs (Figure 3B), including 1470 up-regulated genes and 1440 down-regulated genes. P3 vs. P4 has 1635 DEGs, of which 874 are up-regulated and 761 are down-regulated (Figure 3B). P2 vs. P4 has 2601 DEGs, of which 1454 are up-regulated and 1147 are down-regulated (Supplementary Figure S2). Considering that the ontogeny level of quail in Q2 is closer to that of pigeon in P3, we next focused on comparing Q2 vs. Q3 and P3 vs. P4. A total of 409 common DEGs were screened (Figure 3C). GO enrichment analysis was conducted to further understand the function of the shared DEGs (Figure 3D). These DEGs were assigned to different GO terms, which were classified into three major categories, including Biological Process (BP), Cellular Component (CC), and Molecular Function (ME). Extracellular, cell adhesion and ossification belong to BP. Membrane and proteinaceous extracellular matrix belong to CC. Binding belongs to ME. In addition, the common DEGs were mapped to some KEGG pathways (Figure 3E). These KEGG pathways were classified into five distinct categories, including Organismal systems, Environmental information processing, Cellular processes, Metabolism, and Diseases.

### Gene Expression Pattern Analysis

**FIGURE 4:**
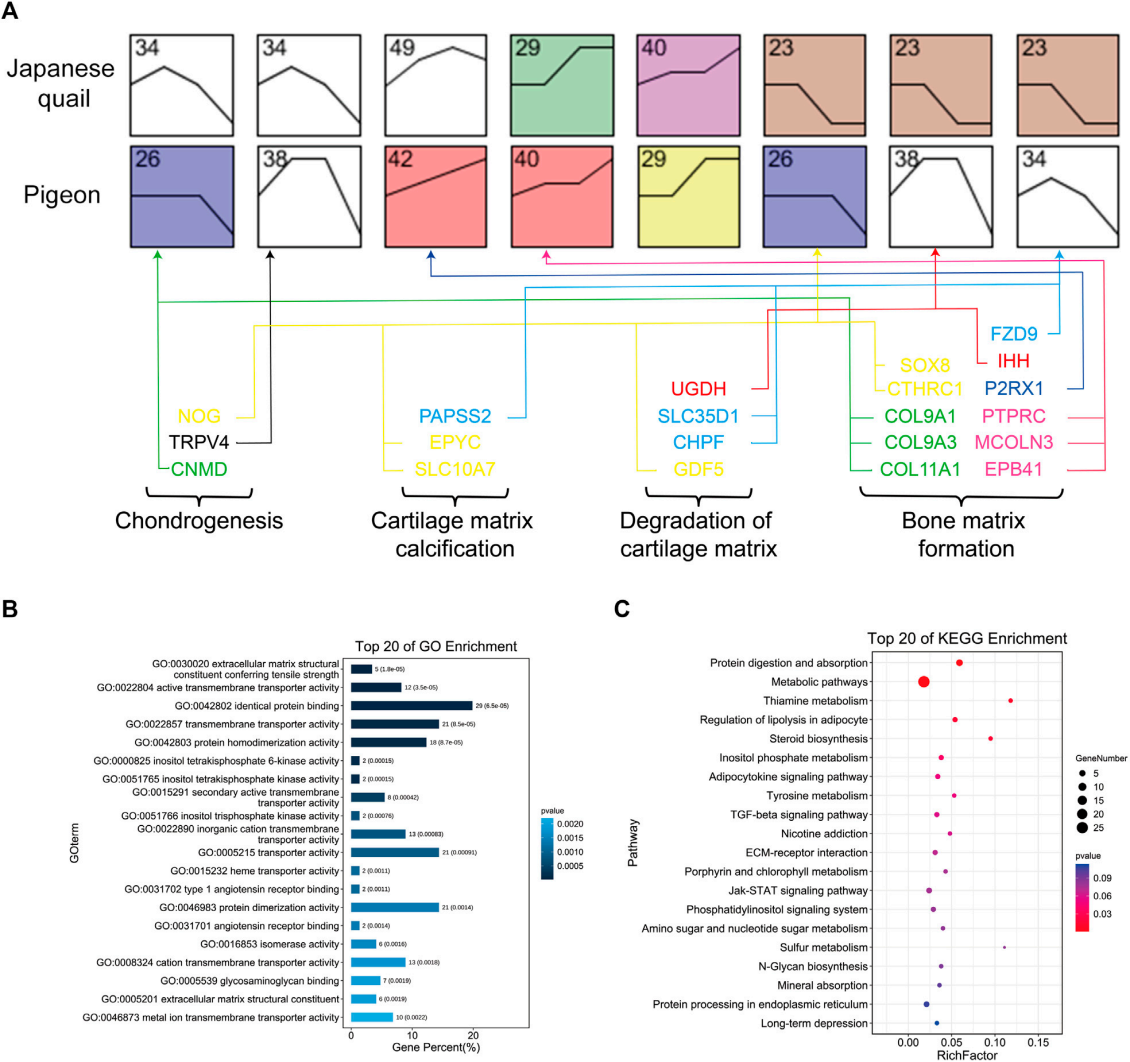
Gene expression pattern analysis of quail and pigeon. **(A)** Expression profile pairs for comparison between quail and pigeons. The number represents the number of the expression profile. The four developmental stages from left to right are E12 (E14), E15 (E17), D1, and D14 of quail (pigeon). The vertical axis represents the gene expression level. Colored boxes indicate that the *p*-value is less than 0.05. **(B)** GO enrichment of genes obtained from the comparison of expression profiles. **(C)** KEGG pathway enrichment of genes obtained from the comparison of expression profiles.

We speculated that the difference in tibia tissue development may be due to the heterochronism of certain gene expressions. To study the tibia gene expression patterns of quail and pigeons before and post-hatching, we screened the genes that were expressed at all four developmental stages, and used STEM software to analyze the gene expression pattern (Supplementary Figure S3) (Ernst and Bar-Joseph, 2006). We selected quail and pigeon expression profiles that conform to this rule and compared them in pairs. A total of eight pairs of expression profiles were obtained, which were Q34 vs. P26 (profile 34 of quail and profile 26 of pigeon, the same below), Q34 vs. P38, Q49 vs. P42, Q29 vs. P40, Q40 vs. P29, Q23 vs. P26, Q23 vs. P38 and Q23 vs. P34 (Figure 4A). We screened the common genes in each pair of the expression profiles and obtained a gene set consisting of 152 genes. GO and KEGG analysis was performed on these genes. GO enriched several biological processes related to skeletal development, such as Proteoglycan metabolic process, Ossification, Embryonic morphogenesis, and Limb morphogenesis (Figure 4B). KEGG pathway analysis enriched to TGF-beta signaling pathway, Protein digestion and absorption, Mineral absorption, and ECM-receptor interaction, which are related to the formation of extracellular bone matrix and bone calcification processes (Figure 4C).

We further compared the 409 DEGs with the 152 genes obtained from the expression profile pairs, and there were 79 genes shared by both said (Supplementary Figure S4). We performed GO analysis of these genes, and 21 genes were involved in the process of endochondral ossification (Figure 4, Supplementary Table S5), including Chondrogenesis, Cartilage matrix calcification, Degradation of cartilage matrix, and Bone matrix formation. These genes include the *COL* gene family (*COL11A1, COL9A3, COL9A1*)*, IHH, MSX2, SFRP1, ATP6V1B1, SRGN, CTHRC1, NOG, GDF5* and so on, which were the candidate genes for the difference of tibial development between pigeon and quail.

### Expression and Phylogenetic Analysis of the *COL* Gene Family

**FIGURE 5:**
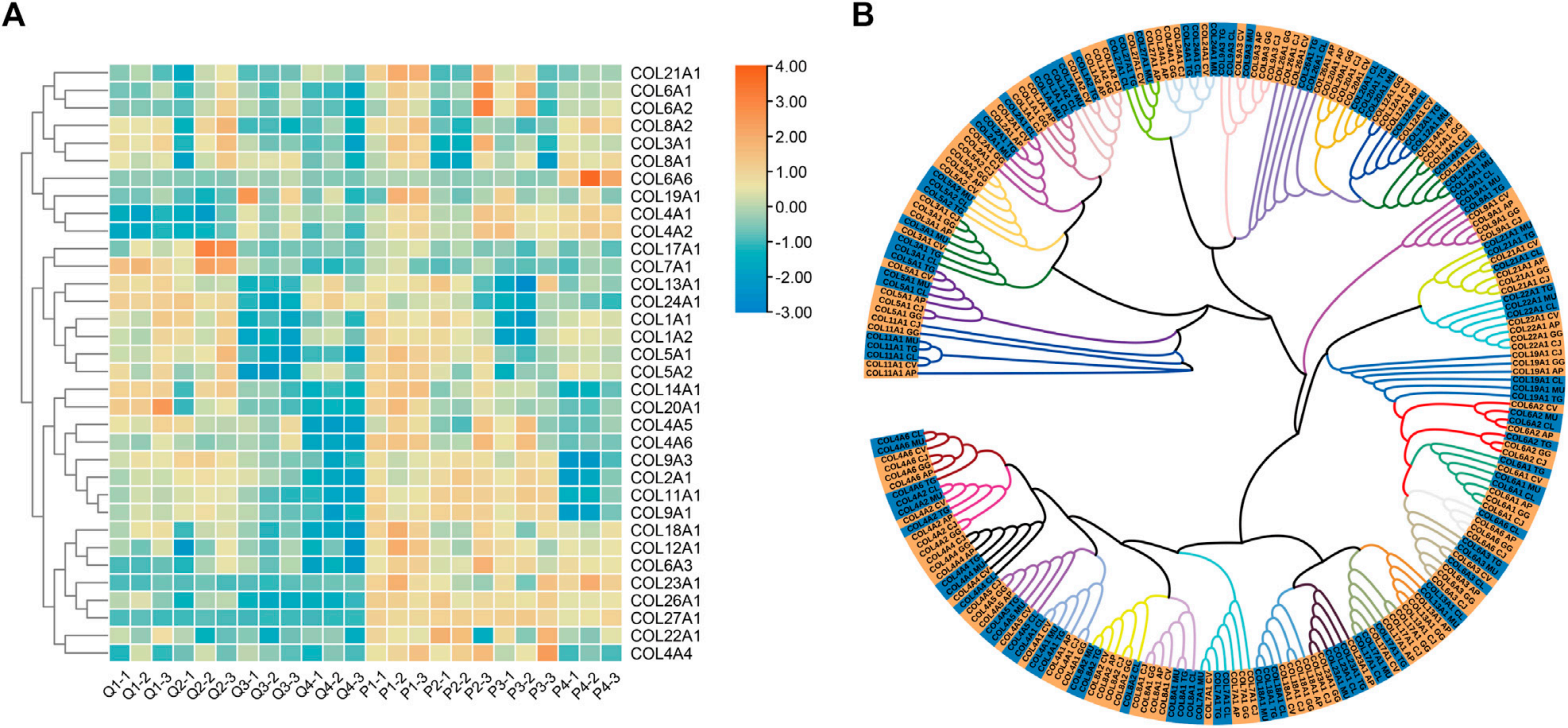
Expression and Phylogenetic Analysis of the *COL* Gene Family. **(A)** The heat map of gene expression in the four growth stages of quails and pigeons. The expression level used in the heat map was log scale of FPKM value. **(B)** Phylogenetic tree of *COL* Gene family. Each color branch represents a gene, and the yellow and blue highlights at the end of the branch are precocial and altricial bird species, respectively. AP: *Anas platyrhynchos* (Duck), CJ: *Coturnix japonica* (Japanese quail), GG: *Gallus gallus* (Chicken), CV: *Charadrius vociferus* (Killdeer), CL: *Columba livia* (Pigeon), MU: *Melopsittacus undulatus* (Budgerigar), TG: *Taeniopygia guttata* (Zebra finch).

*COL9A1, COL9A3,* and *COL11A1* were consistently highly expressed in the tibia tissues at the four growth stages of quail and pigeon, and the sequence of expression time was different between the two species (Figure 4A). Therefore, we focused on the *COL* gene family and analyzed its evolutionary relationship in precocial and altricial birds. A total of 34 genes of the gene family were both expressed in quail and pigeon. Protein sequences of these genes were downloaded from NCBI (Supplementary Table S2). We analyzed the expression of these genes at different stages in pigeons and quails (Figure 5A). It is found that there are obvious differences in gene expression trends, and most genes show a tendency to delay expression in pigeons. *COL*-proteins Maximum Likelihood tree was constructed using seven species, among which Japanese quail, Duck, Chicken, and killdeer belong to precocial birds; Pigeon, Budgerigar, and Zebra finch belong to altricial birds (Figure 5B). The seven species showed a clear tendency to cluster according to precocialism and altricialism on the phylogenetic tree branches of most genes. But killdeer is an exception, which was clustered with altricial birds in many genes (Figure 5B). We found that there was a certain correlation between the expression clustering and phylogenetic clustering of many genes in the *COL* family. For example, *COL1A1* and *COL1A2, COL6A1 and COL6A2* were clustered together in both analyses (Figures 5A,B). It suggested that the *COL* gene family may play a role in the differences in developmental patterns of some precocial and altricial bird species.

## Discussion

Factors that affect the hatching time of bird’s eggs include weight, egg storage temperature, incubation humidity, and rotation, among others (Alsattar and Hassan, 2015; Iqbal et al., 2016; Belnap et al., 2019). White king pigeon (*Columba*) and Japanese quail (*Coturnix japonica*) are typical altricial and precocial species, respectively. Not only do they have similar body size, but their eggs are similar in size and incubation period, so these two species are suitable for comparison. Here we choose eggs of similar size within the species and tried to keep them in an environment as consistent as possible, including storage temperature and incubation humidity. Under the incubation conditions of this experiment, the incubation period of each batch is stable, which is conducive to the comparison between species.

Comparative embryology is the empirical basis for discovering how developmental processes have evolved (Li et al., 2019). Avian embryology has traditionally been biased towards the embryology of precocial birds (Ainsworth et al., 2010; Nagai et al., 2011). Only a few comparative studies of early embryonic development of precocial and altricial bird species can be found (Blom and Lilja, 2005; Olea and Sandoval, 2012). Our research compared the embryonic development patterns of quail and pigeon. The quail embryos grew faster in the early phase and developed more maturely when hatching (Figures 1A,D). However, the early growth rate post-hatching of pigeons is significantly faster than quails (Figures 1B,C). This is consistent with precocial and altricial bird species (Ainsworth et al., 2010; Murray et al., 2013; Carril and Tambussi, 2015; Olea B et al., 2016). Some studies show that altricial species are characterized by the rapid early development of “supply” organs, such as digestive organs. In contrast, precocial species exhibit a more rapid early growth of other “demand” organs, such as the brain, muscles, skeleton, and feathers (Charvet and Striedter, 2011; Chen et al., 2016; Augustine et al., 2019; Zhou et al., 2019).

The results of embryonic skeleton staining (Figure 1D) and the analysis of tibia tissue morphology (Figure 2) revealed that the different skeletal development speeds during hatching, which characterize precocial and altricial neonates, were associated with different patterns of embryonic development in the Japanese quail and pigeon. Heterochronies have been found in precocial and altricial species, but the overall ossification sequence pattern is relatively conserved compared to variations in other developmental characters, although the variability is likely to differ among taxa and character sets (Prochel et al., 2008; Werneburg et al., 2009). The mechanical performance of limb bones is closely associated with an animal’s locomotor capability. The Japanese quail is a typical ground-dwelling precocial bird, hatchlings have well-developed hind limbs (Nakane and Tsudzuki, 2010). Pigeons use hind limbs to dig and forage, but their primary locomotor pattern is flying. There are differences in the geometrical and mechanical characteristics of the femur of hatchling quails and pigeons, hatchling quails had stiffer bone tissues (Wei and Zhang, 2019). The mechanical performance of femora exhibited negative allometry in quails but positive allometry in pigeons, which is related to the precocial and altricial species’ decreasing and increasing functional demands for hind limbs during growth, respectively (Pourlis et al., 1998; Gillian, 2000; Ahmed and Soliman, 2013).

We utilized the comparative transcriptome approach to screen some pathways and genes related to the heterochronism of tibia development between Japanese quail and pigeon. The pathways were mainly associated with the formation of extracellular bone matrix and the process of bone calcification, such as ECM-receptor interaction, Protein digestion and absorption, and TGF-β signaling pathway. TGF-β signaling pathway regulates cell proliferation, apoptosis, differentiation, and migration (Chang et al., 2002). The calcification of the limb bones of vertebrates is endochondral ossification, which is a complex physiological process (Shawn et al., 2019). The process is accompanied by the proliferation and apoptosis of chondrocytes, the formation and degradation of cartilage matrix and collagen, and finally, the formation of calcified bone matrix (Mackie et al., 2008; Ishijima et al., 2012). The extracellular matrix (ECM) consists of a complex structure, including the cartilage matrix and bone matrix, and serves a vital role in tissue and organ morphogenesis (Milner and Campbell, 2002). The cartilage matrix and bone matrix contain various collagen secreted by hypertrophic chondrocytes and osteoblasts (Mark and Zhou, 2016). Transmembrane molecules mediate specific interactions between cells and the ECM. These interactions lead to direct or indirect control of cellular activities such as adhesion, migration, differentiation, proliferation, and apoptosis (Bosman and Stamenkovic, 2003).

Gene *COL9A1*, *COL9A3*, and *COL11A1* belong to the pathway of ECM-receptor interaction, Protein digestion, and absorption. The collagen gene family is a scattered gene family, which plays a vital role in protein synthesis and skeleton development (Ottani et al., 2001). Homozygous Type IX collagen variants (*COL9A1*, *COL9A2*, and *COL9A3*) causing recessive Stickler syndrome (Nixon et al., 2019). *COL9A1* and *SOX9* are related to the genetic susceptibility of postmenopausal osteoporosis (Hongliang et al., 2020). Genetic mutations of *COL2A1* and *COL11A1* seen in Stickler Syndrome affect the incidence of mandibular distraction osteogenesis (Swanson et al., 2021).

Birds are a very large family, with more than 10,000 members (MacArthur et al., 1961). Precocial and altricial bird species are not completely separated in evolutionary relationship (Hackett et al., 2008). For example, the killdeer used in our research belongs to Charadriiformes, whose members are precocial but are more closely related to Pigeon, Budgerigar, and Zebra finch (Prum et al., 2015). This may be related to their specific living environment, reflecting the complexity and species specificity of the evolutionary relationship between precocial and altricial bird species. In the phylogenetic tree of the COL gene family, Japanese quail, Chicken and Duck cluster together, while Killdeer clustered with altricial birds in many genes. It shows that genetic relationships may also play a role in it. We speculate that the developmental regulation mechanism of different precocial birds may be different. In other words, the candidate genes screened in this study are related to different developmental patterns of tibia in quail and pigeon, but these genes may not play a same significant role in other precocial and altricial bird species. More research is needed to verify it.

Whether it has the ability of locomotion at hatching is one of the developmental strategies of precocial and altricial birds, and it is a complicated biological problem. This research focused on skeletal development and finally screened 21 genes, including some members of the *COL* gene family. These genes affect the locomotor ability of hatchlings of the quail and pigeon by regulating the tibia development. In the future, it is necessary to carry out more work to conduct in-depth research on this issue, such as cross-species comparative genomics and multi-tissue analysis covering nerves and muscles.

## Data Availability

The data presented in the study are deposited in the Genome Sequence Archive (GSA) repository (https://ngdc.cncb.ac.cn/gsa/), accession number was CRA005066.
