# Supplementary material for: Developmental Transcriptome Profiling of the Tibial Reveals the Underlying Molecular Basis for Why Newly Hatched Quails Can Walk While Newly Hatched Pigeons Cannot
Source: Front Cell Dev Biol. 2022 Feb 7;10:745129. doi: 10.3389/fcell.2022.745129 (PMC8858812; doi:10.3389/fcell.2022.745129)
Supplement: Supplementary file 1 [file DataSheet1.pdf]

Table S1 Embryo skeletal staining method from 10-day embryos to hatching

Step Number	Brief description of the method
Step 1	Remove skin, viscera, and adipose tissue. Fix with 95% alcohol for 4-6 days until the embryo is hard to touch.
Step 2	Fix and stain for 1 day at room temperature in a freshly prepared solution of 95% ethanol (80 ml), 20 ml acetic acid, and 15 mg Alcian blue 8GX.
Step 3	Dehydrate in 70% ethanol for 1 day.
Step 4	Stain and macerate for 12-24 hours in a freshly prepared solution of 70% ethanol (400 ml) and 20 mg alizarin red S.
Step 5	Soak in distilled water for a while, and then macerate for 2-7day in 1% KOH.
Step 6	Clear in glycerine / H2O solutions of increasing concentration (25, 50, and 75%) of glycerine for 7 days each, to 100% glycerine for storage.

Table S2. The NCBI reference protein sequence numbers of 34 genes in phylogenetic analysis.

Gene name	*Anas platyrhynchos*	*Coturnix japonica*	*Gallus gallus*	*Charadrius vociferus*	*Columba livia*	*Melopsittacus undulatus*	*Taeniopygia guttata*
COL11A1	XP_038038616.1	XP_015725394.1	XP_040533585.1	XP_009883081.1	XP_021149154.1	XP_030910622.1	XP_030135131.3
COL12A1	XP_038033649.1	XP_032299328.1	XP_015140225.1	XP_009894059.1	XP_005516028.2	XP_030908617.2	XP_041571000.1
COL13A1	XP_038037456.1	XP_032301192.1	XP_040559144.1	/	XP_021148564.1	XP_033918066.1	/
COL14A1	XP_038031226.1	XP_015711673.1	XP_040532787.1	XP_009879337.1	XP_021149914.1	XP_033917219.1	XP_030122477.3
COL17A1	XP_027315684.2	XP_015722519.1	NP_001292131.1	XP_009879845.1	XP_021151543.1	XP_033918581.1	XP_030132216.3
COL18A1	XP_038037851.1	XP_015723177.1	NP_989495.1	XP_009889710.1	XP_013226958.1	XP_033921078.1	XP_030132844.3
COL19A1	XP_027310837.2	XP_015714626.1	XP_004940506.1	/	XP_013223881.1	XP_033916810.1	XP_041570983.1
COL1A1	XP_038024652.1	XP_015741354.1	XP_024999899.1	/	XP_021136665.1	XP_033926254.1	/
COL1A2	XP_038029840.1	XP_015709029.1	NP_001073182.2	XP_009893653.1	XP_005504983.1	XP_033918038.1	XP_032602559.1
COL20A1	XP_038022205.1	XP_015737180.1	NP_001004392.2	XP_009891877.1	XP_021139481.1	XP_033922804.1	XP_030144588.3
COL21A1	XP_038033338.1	XP_015714716.1	XP_004940520.2	XP_009878135.1	XP_021149165.1	XP_005153247.3	XP_030123227.3
COL22A1	XP_038031001.1	XP_032298804.1	NP_001132911.1	XP_009886476.1	XP_021154543.1	XP_005145553.3	XP_030122605.3
COL23A1	XP_038042584.1	XP_032303275.1	XP_040503131.1	/	XP_021144554.1	XP_030903303.2	XP_041574872.1
COL24A1	XP_027318814.2	XP_015725661.1	XP_040560780.1	XP_009883186.1	XP_021149887.1	XP_005148161.2	XP_030134959.3
COL26A1	XP_038021677.1	XP_015736502.1	XP_040506282.1	XP_009883420.1	XP_021142672.1	/	XP_030143804.3
COL27A1	XP_027326476.2	XP_032304017.1	XP_025011860.2	XP_009886342.1	XP_021137799.1	XP_033923246.1	XP_030142347.3
COL2A1	XP_038028181.1	XP_015705870.1	NP_989757.1	XP_009891367.1	XP_021138352.1	XP_033927528.1	XP_032600780.2
COL3A1	XP_027317808.1	XP_015723325.1	NP_990711.2	XP_009888519.1	XP_013226117.1	XP_030901733.1	XP_030133720.3
COL4A1	XP_027300111.2	XP_015706908.1	NP_001155871.1	XP_009890461.1	XP_013224666.1	XP_033916098.1	XP_030136204.1
COL4A2	XP_027300107.2	XP_015706907.1	XP_015133342.3	XP_009890460.1	XP_021152299.1	XP_030901418.2	XP_030136192.1
COL4A4	XP_038039909.1	XP_015726876.1	XP_040535220.1	XP_009888569.1	XP_021147804.1	XP_012983997.1	XP_030135972.3
COL4A5	XP_027321233.1	XP_015715963.1	XP_015134091.2	XP_009887593.1	XP_021137991.1	XP_033919414.1	XP_030128159.3
COL4A6	XP_038040586.1	XP_015715966.1	XP_015134089.2	XP_009887653.1	XP_021152457.1	XP_033919764.1	XP_030128163.3
COL5A1	XP_038021077.1	XP_015734782.1	NP_990121.2	XP_009890627.1	XP_021135674.1	XP_033923569.1	XP_041575618.1
COL5A2	XP_038038075.1	XP_015723324.1	XP_004942453.2	XP_009888430.1	XP_005513139.1	XP_005145648.1	XP_002191994.5
COL6A1	XP_005024802.2	XP_015723180.1	XP_040559290.1	XP_009889695.1	XP_005515324.1	XP_033921256.1	XP_002190865.3
COL6A2	XP_005024800.3	XP_015723179.1	XP_015144623.1	XP_009889694.1	XP_005515325.1	XP_033921169.1	XP_030132882.3
COL6A3	XP_038038178.1	XP_015723565.1	XP_040559308.1	XP_009885888.1	XP_021142382.1	XP_033921540.1	XP_030132911.3
COL6A6	XP_038031064.1	XP_015709514.1	XP_040518766.1	/	XP_021139209.1	/	/
COL7A1	XP_038041693.1	XP_015730557.1	XP_015148859.2	XP_009888708.1	XP_021153665.1	XP_033922440.1	XP_041574708.1
COL8A1	XP_038038307.1	XP_032303562.1	XP_015151511.1	XP_009889922.1	XP_021155400.1	XP_033930283.1	XP_032602561.1
COL8A2	XP_038023107.1	XP_015738726.1	XP_425780.4	XP_009893908.1	XP_021153161.1	XP_033925051.1	XP_030146355.3
COL9A1	XP_038032031.1	XP_015714611.1	XP_040553266.1	XP_009878824.1	XP_021149162.1	XP_005153232.1	XP_030123246.3
COL9A3	XP_005029067.2	XP_015737083.1	NP_990636.1	XP_009891753.1	XP_005499645.1	XP_030902894.2	XP_030144277.3

Table S3 Data evaluation table for transcriptome samples

Smaples	Clean Reads	Obtained Base (bp)	Q20 (%)	Q30 (%)	GC (%)
P1-1	23,081,365	6,859,201,918	97.86	93.94	49.82
P1-2	21,519,111	6,426,477,766	97.73	93.68	49.2
P1-3	20,295,609	6,042,407,434	97.79	93.84	49.19
P2-1	20,678,552	6,149,168,566	97.8	93.85	49.77
P2-2	23,237,701	6,907,193,428	97.73	93.72	50.64
P2-3	25,691,255	7,630,171,532	97.77	93.76	50.16
P3-1	20,491,791	6,119,937,024	97.69	93.64	48.97
P3-2	21,381,044	6,355,318,010	97.86	93.94	48.16
P3-3	23,959,184	7,106,029,776	97.67	93.57	49.35
P4-1	22,910,685	6,842,300,968	97.85	93.88	49.32
P4-2	23,024,409	6,850,481,210	97.83	93.89	50.45
P4-3	21,069,383	6,281,350,280	97.76	93.75	50.27
Q1-1	19,192,671	5,712,843,058	97.88	94.04	50.91
Q1-2	22,140,375	6,597,654,816	97.57	93.39	49.63
Q1-3	20,014,153	5,974,963,290	97.48	93.28	49.96
Q2-1	26,075,343	7,777,351,712	97.69	93.72	50.58
Q2-2	24,629,117	7,336,593,074	97.65	93.63	50.1
Q2-3	19,763,224	5,863,385,960	97.47	93.29	50.32
Q3-1	25,377,709	7,566,235,298	97.73	93.71	50.28
Q3-2	22,248,136	6,623,768,642	97.69	93.69	50.74
Q3-3	21,720,333	6,475,066,908	97.81	93.83	49.96
Q4-1	26,152,136	7,818,586,094	97.65	93.53	48.25
Q4-2	21,383,383	6,376,033,914	97.84	94	49.58
Q4-3	21,913,816	6,547,959,760	97.88	94.05	49.37

Obtained Base: Clean Reads multiplied by sequence length; Q20: The percentage of bases with a mass value of 20 or greater; Q30: The percentage of bases with a mass value of 20 or greater; GC: Content of GC base in the sample.

Table S4. NT comparison results

Smaples	Align%	Hit1	Hit1 Percent (%)	Hit2	Hit2 Percent (%)
P1-1	96.15	Columba livia	71.08	Streptopelia turtur	14.24
P1-2	96.4	Columba livia	72.14	Streptopelia turtur	13.07
P1-3	95.2	Columba livia	73.1	Streptopelia turtur	12.81
P2-1	96.85	Columba livia	74.13	Streptopelia turtur	12.44
P2-2	96.75	Columba livia	73.9	Streptopelia turtur	11.52
P2-3	96.95	Columba livia	71.17	Streptopelia turtur	12.63
P3-1	96.65	Columba livia	75.68	Streptopelia turtur	12
P3-2	96.7	Columba livia	73.31	Streptopelia turtur	12.77
P3-3	96.85	Columba livia	74.65	Streptopelia turtur	11.82
P4-1	96.25	Columba livia	76.62	Streptopelia turtur	12.88
P4-2	95.85	Columba livia	74.9	Streptopelia turtur	11.94
P4-3	95.95	Columba livia	74.72	Streptopelia turtur	12.08
Q1-1	96.35	Coturnix japonica	77.68	Gallus gallus	6.69
Q1-2	95	Coturnix japonica	85.68	Gallus gallus	4.26
Q1-3	96.75	Coturnix japonica	88.11	Gallus gallus	2.63
Q2-1	94.9	Coturnix japonica	87.98	Gallus gallus	3.16
Q2-2	94.6	Coturnix japonica	87.57	Gallus gallus	3.69
Q2-3	96.35	Coturnix japonica	90.08	Gallus gallus	2.23
Q3-1	95.65	Coturnix japonica	85.15	Gallus gallus	4.12
Q3-2	96.4	Coturnix japonica	88.74	Gallus gallus	3.42
Q3-3	96.95	Coturnix japonica	85.09	Gallus gallus	4.48
Q4-1	96.4	Coturnix japonica	89.1	Gallus gallus	2.33
Q4-2	94.65	Coturnix japonica	88.64	Gallus gallus	2.79
Q4-3	96.75	Coturnix japonica	88.16	Gallus gallus	2.79

Align: Comparison efficiency of sample and NT library; Hit1: Compare to the first species; Hit1 Percent: Comparison efficiency of the first species; Hit2: Compare to the second species; Hit2 Percent: Comparison efficiency of the second species.

Table S5 Genes involved in the process of endochondral ossification

Processes of endochondral ossification	Brief description of the processes	Related genes	GO ID
Chondrogenesis	Mesenchymal cells differentiate into chondrocytes. Proliferating chondrocytes produce many matrix molecules, such as collagen II and aggrecan.	*CNMD NOG TRPV4*	GO:0051216 GO:0060351
Cartilage matrix calcification	Proliferating chondrocytes differentiate into collagen X-expressing hypertrophic chondrocytes, which secret alkaline phosphatase. Cartilage matrix mineralization, hypertrophic chondrocytes then undergoes apoptotic.	*EPYC PAPSS2 SLC10A7*	GO:0060348
Degradation of cartilage matrix	Vascular invasion, osteoclasts degrade and absorb mineralized cartilage matrix.	*UGDH SLC35D1 CHPF GDF5*	GO:0030206 GO:0048706 GO:0030509
Bone matrix formation	Osteoblasts secrete collagen I and form a bone matrix.	*IHH SOX8 FZD9 PTPRC MCOLN3 P2RX1 EPB41 CTHRC1 COL11A1 COL9A3 COL9A1*	GO:0001649 GO:0001503 GO:0051209 GO:0051924 GO:0005201

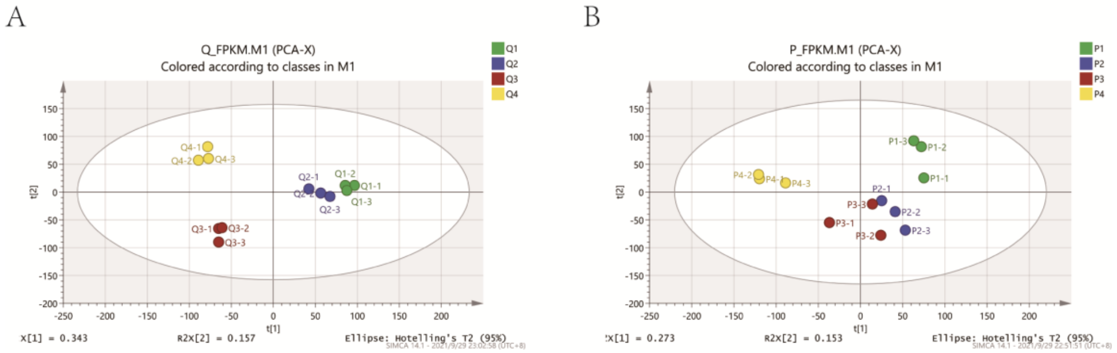

Figure S1 Principal component analysis of transcriptome data of pigeon and quail.

Q: quail; P: pigeon.

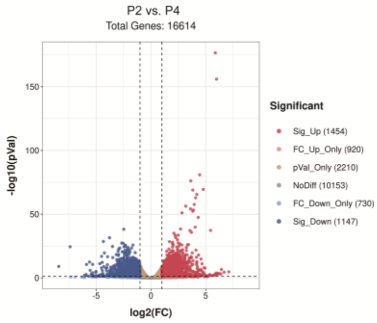

Figure S2 Volcano plots of genes in group P2 vs. P4.

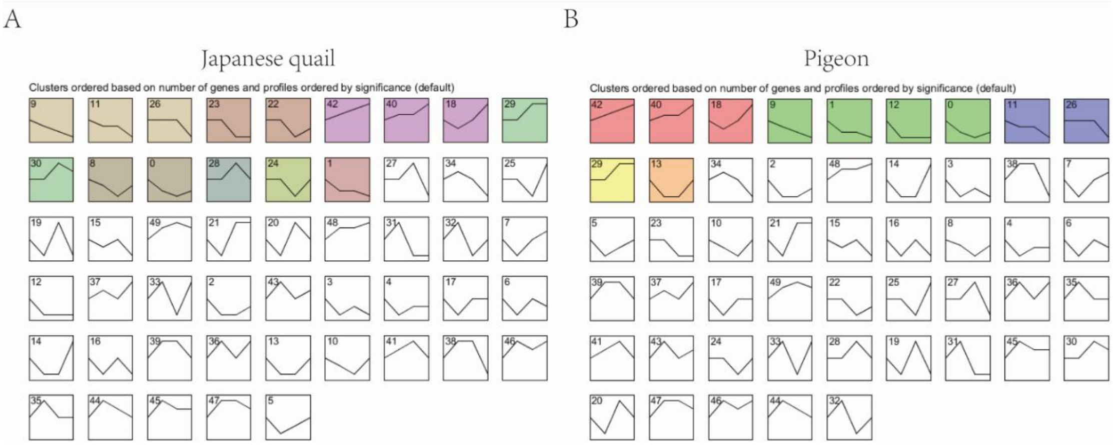

Figure S3 Gene expression pattern analysis of quail and pigeon. **(A)** The pattern of quail. **(B)** The pattern of pigeon.

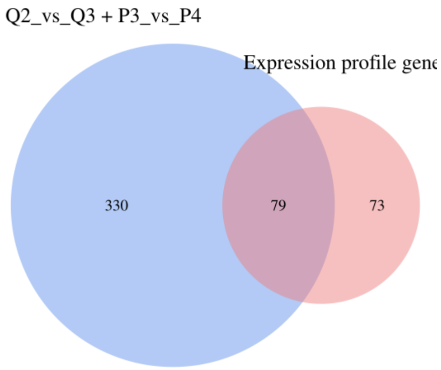

Figure S4 Common genes between 409 DEGs and the 152 genes obtained from the expression profile pairs.
